# Non-Polio Enteroviruses from Acute Flaccid Paralysis Surveillance in Korea, 2012–2019

**DOI:** 10.3390/v13030411

**Published:** 2021-03-05

**Authors:** Youngsil Yoon, Yong-Pyo Lee, Deog-Yong Lee, Hye-Jin Kim, June-Woo Lee, Sangwon Lee, Chun Kang, Wooyoung Choi, Joong Hyun Bin, Young Hoon Kim, Myung-Guk Han, Hae Ji Kang

**Affiliations:** 1 Division of Viral Diseases, Korea Disease Control and Prevention Agency, Cheongju 28159, Korea; sjdtlf1220@korea.kr (Y.Y.); leedy0610@korea.kr (D.-Y.L.); neofrontier@korea.kr (H.-J.K.); epilsw@korea.kr (S.L.); ckang59@gmail.com (C.K.); mghan@korea.kr (M.-G.H.); 2 Division of Infectious Disease Diagnosis Control, Regional Centers for Disease Control and Prevention, Korea~Disease Control and Prevention Agency, Cheongju 28159, Korea; lyp0112@korea.kr (Y.-P.L.); wychoi65@korea.kr (W.C.); 3 Division of Vaccine Clinical Research, National Institute of Health, Cheongju 28159, Korea; junewoo1213@korea.kr; 4 College of Medicine, Catholic University of Korea, Seoul 06591, Korea; 37class@daum.net (J.H.B.); pedkyh@catholic.ac.kr (Y.H.K.)

**Keywords:** acute flaccid paralysis (AFP), surveillance, poliovirus, enterovirus

## Abstract

The risk of polio importation and re-emergence persists since epidemic polio still occurs in some countries, and the resurgence of polio occurring almost 20 years after polio eradication was declared in Asia has been reported. We analyzed the results of acute flaccid paralysis (AFP) surveillance in Korea to assess the quality of AFP surveillance and understand the etiology of non-polio enterovirus (NPEV)-associated central nervous system diseases in a polio-free area. We investigated 637 AFP patients under 15 years of age whose cases were confirmed during 2012–2019 by virus isolation, real-time reverse transcription polymerase chain reaction, and VP1 gene sequencing. Among the 637 AFP cases, NPEV was detected in 213 (33.4%) patients, with the majority observed in EV-A71, with 54.9% of NPEV positives. EV-A71 has been shown to play a role as a major causative agent in most neurological diseases except for Guillain-Barré syndrome (GBS), acute disseminated encephalomyelitis (ADEM), and meningitis. This study provides information on the AFP surveillance situation in Korea and highlights the polio eradication stage in the monitoring and characterization of NPEV against the outbreak of neurological infectious diseases such as polio.

## 1. Introduction

Poliomyelitis is a highly contagious vaccine-preventable disease caused by the poliovirus. Since the polio vaccine was developed in the 1950s, global polio eradication activities such as the Global Polio Eradication Initiative (GPEI), including immunization programs for polio, have reduced the global incidence by over 99% since 1988 from an estimated 350,000 cases to 33 cases in 2018 [1]. Two out of three wild poliovirus strains have been eradicated worldwide; eradication of wild poliovirus type 2 and type 3 was declared in 2015 and 2019, respectively [2].

In Korea, the inactivated polio vaccine (IPV) and oral poliovirus vaccine (OPV) became available in 1958 and 1962, respectively [3]. The polio vaccine was introduced to the national immunization program (NIP) in 1983, and a four-dose polio vaccine schedule was recommended at 2, 4, and 6 months and at 4–6 years of age [3]. Due to the risk of vaccine-associated paralytic poliomyelitis and vaccine-derived polioviruses, OPV was withdrawn from the NIP in 2004 [4].

A nationwide acute flaccid paralysis (AFP) surveillance has been conducted by the Korea Centers for Disease Control and Prevention (KCDC) in Korea since 1998 as part of the GPEI for detecting, reporting, and investigating suspected cases of poliomyelitis [5]. The World Health Organization (WHO) recommended several indicators to evaluate the quality of the surveillance system. These indicators include the annualized non-polio AFP rate (target: non-polio AFP rate of ≥1 case per 100,000 children under 15 years of age) and the completeness of investigation (target: two adequate stool specimens collected 24–48 h apart and within 14 days after symptom onset, from ≥80% of AFP cases) [6,7]. As a result of the national activities, including the routine immunization program and monitoring of polio by the national surveillance system to control polio, no cases of infection with the wild poliovirus have been reported since 1984, and polio eradication was declared in 2000 [5].

This paper evaluated the AFP surveillance system and analyzed the outcomes during 2012–2019 to improve the AFP surveillance system and maintain a polio-free state in Korea.

## 2. Materials and Methods

The AFP surveillance system conducted by the Korea Disease Control and Prevention Agency (KDCA) has been used to monitor and detect the presence of circulating wild poliovirus since 1998 in Korea. A total of 50 nationwide hospitals have participated in the AFP surveillance system, and at the participating hospitals, specimens are collected by the pediatric neurologists from patients with AFP (including Guillain-Barré syndrome) and sent to the KDCA. According to the WHO polio laboratory manual, two adequate stool samples should be collected 24–48 h apart within 14 days of onset of symptoms [7]. The patients’ information (demographics, clinical history, vaccination history, and dates of stool specimen collection) was recorded. After the onset of symptoms, a 60-day follow-up report was used to identify the signs of residual paralysis in all the patients.

### 2.1. Cell Culture

The conventional tube cell culture method was used in line with the WHO polio laboratory manual for poliovirus cell culture [5,7]. The stool sample was made into a suspension with 10 mL phosphate buffer solution, 1 mL chloroform, and 1 g of glass beads. The mixture was vigorously shaken for 20 min and centrifuged at 1500× *g* for 20 min. Two cell culture tubes containing rhabdomyosarcoma (RD-A) cells or murine (L20B) cells in 1 mL of maintenance medium were inoculated with 0.2 mL of specimen extract. The tubes were examined daily for evidence of cytopathic effect (CPE). When CPE above 3+ (indicating the percentage of cells affected: 1+, 2+, 3+, and 4+ indicate up to 25%; 25–50%; 50–75%, and 75–100%, respectively) was obtained, the infected cells were harvested and kept frozen at −20 °C.

### 2.2. Virus Detection

Viral RNA was extracted from the specimens using a QIAamp viral RNA Mini Kit (Qiagen, Hilden, Germany) according to the manufacturer’s instructions. One-step real-time reverse transcription polymerase chain reaction (RT-PCR) was performed using a 2X qScript XLT one-step quantitative RT-PCR (RT-qPCR) Toughmix (Quanta Biosciences, Beverly, MA, USA) and the pan-enterovirus specificity primer and probe [8]. The conventional RT-PCR for the amplification of the enterovirus (EV) VP1 region was performed by using an EV VP1 detection kit (iNtRon Biotechnology, Gyeonggi, Korea). This EV detection kit that amplifies the region encoding the enterovirus partial VP1 N-terminus to characterize the virus strains was used in nested RT-PCRs and the expected size of the amplicon was 350~371 bp. To determine the non-polio EV (NPEV) genotype, all positive RT-PCR products were sequenced directly and compared for sequence homology with the NPEV sequences available in GenBank using BLAST (http://blast.ncbi.nlm.nih.gov/Blast.cgi, accessed on 1 December 2020).

### 2.3. Ethical Statement

The study was approved by the Institutional Review Board of the Catholic University of Korea (approval no. UC12SNM10022H).

### 2.4. Statistical Analysis

Data analysis was performed using SAS 9.1 software (SAS Institute Inc., Cary, NC, USA). Comparisons between groups were conducted using chi-square and Fisher’s exact tests for categorical variables. A *p*-value < 0.05 was considered statistically significant for all the analyses.

## 3. Results

The total number of AFP patients was 637, and annually, 80 cases were reported during 2012–2019. The non-polio AFP rate was ≥1.0, and >85% of stool specimens were adequately collected annually. All AFP cases were followed up for 60 days after the onset of paralysis for residual paralysis (Table 1). The regional non-polio AFP rates were highest in Gyeongsangnam-do Province (2.67) and lowest in Gwangju Province (0.05). Only seven of the 17 provinces satisfied the targeted annual non-polio AFP rate of ≥1.0. 

Of the 637 participants in this study (median age, 5 (range < 1–19) years), 371 (58.20%) were male and 266 (41.80%) were female. The male AFP patients had a significantly higher incidence than the female AFP patients (*p* < 0.01). The highest incidence of AFP case occurred among children aged 1–5 years at 41.6 (95% confidence interval (CI), 30.49–52.76) cases annually; followed by that of children aged 6–10 years (19 cases, 95% CI, 14.19–23.81); 11–15 years (14 cases, 95% CI, 11.68–16.32); under 12 months (3.9 cases, 95% CI, 2.06–5.69); and lowest among those aged >15 years (1.1 cases, 95% CI, 0.08–2.17).

AFP patients presented with fever in 64.20% of cases (409/637, *p* = 0.0001). Among the AFP patients, 72.80% had received at least one dose of the polio vaccine, and 24.80% did not know their polio immunization status (Table 2). Overall, we included 637 patients during 2012–2019 for the analysis of virus isolation from RD-A and L20B cells; no poliovirus was detected in both cell lines. A total of 49 NPEV were isolated from the RD-A cells, whereas L20B cells showed CPE after the specimens were inoculated in 2 of 49 positive RD-A cells (Table 3).

Of the 637 AFP patients analyzed using both real-time and conventional RT-PCR, 213 (33.4%) were positive for NPEV. EV-A was most frequently detected (146, 68.5%), especially EV-A71, which was detected in 54.9% of NPEV positives. The highest detection rate for EV of 56.6% (47/83) was observed in 2013, whereas NPEVs were rarely detected in 2017 and 2018 at <10% of AFP cases yearly (Table 4). Among cases that were positive for NPEV RNA, the rate of virus isolation for EV-B of 51.4% (18/35) was higher than for other EV species; EV-A was 17.8% (26/146), and no EV-C was isolated in the cell culture (Table 4).

**Figure 1:**
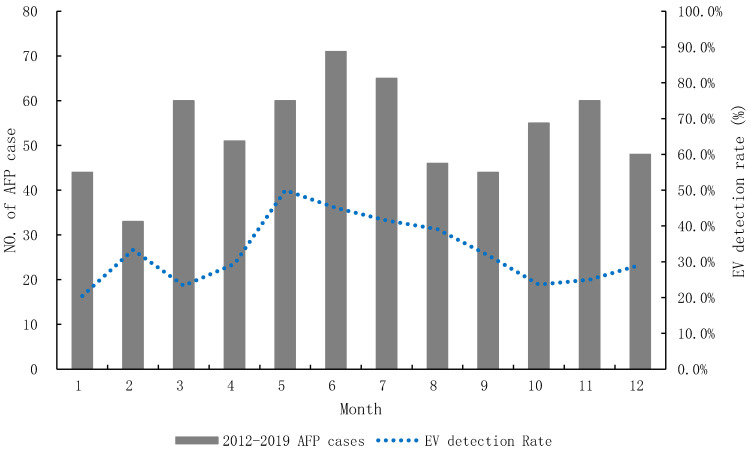
Monthly detection rate of AFP surveillance in Korea, 2012–2019.

The temporal distribution of AFP showed random distribution throughout the year with 53 cases/month (range 33–71 cases/month). The NPEV detection rate of AFP was from 20.5% to 50.0%, and the highest at 50.0% occurred in May (Figure 1).

**Figure 2:**
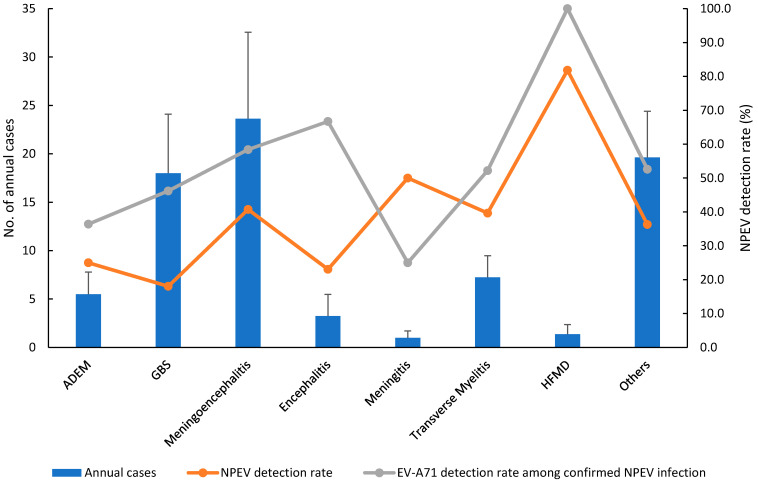
Clinical diagnoses of AFP surveillance system in Korea, 2012–2019. Error bars show the standard deviation of cases during 2012–2019. ADEM, acute disseminated encephalomyelitis; GBS, Guillain-Barré syndrome; HFMD, hand-foot-and-mouth disease; Other, acute respiratory diseases, myocarditis, sepsis neonatorum, etc.; AFP, acute flaccid paralysis; NPEV, non-polio enterovirus.

The bar graph shows the monthly number of AFP patient cases, and the line graph displays the NPEV detection rate in the AFP surveillance system, 2012–2019. The diagnoses of patients with AFP were diverse, including meningoencephalitis reported in 189 cases (29.7%), followed by Guillain-Barré syndrome (GBS) in 144 cases (22.6%), transverse myelitis in 58 cases (9.1%), acute disseminated encephalomyelitis (ADEM) in 44 cases (6.9%), encephalitis in 26 cases (4.1%), hand-foot-mouth diseases (HFMD) in 11 cases (1.7%), meningitis in 8 cases (1.3%), poliomyelitis in 6 cases (0.9%), and other clinical syndromes, including myositis, hypokalemic paralysis, infection, and undefined paralysis in 151 cases (23.7%) (Figure 2). Except for the patient group with GBS, ADEM, and encephalitis, >30% of NPEV detection rates were identified in most of the patient groups (Figure 2). Higher EV-A71 detection rates of >50% occurred in HFMD (100%), encephalitis (66.7%), meningoencephalitis (58.4%), transverse myelitis (52.2%), and others (52.6%) (Figure 2).

## 4. Discussion

Although the incidence of polio globally has decreased and wild poliovirus transmission has been interrupted in many countries, polio epidemics have been and are still being reported in Pakistan and Afghanistan [2]. In 2019, polio re-emerged after almost 20 years in the Philippines and Malaysia after both countries were declared polio-free in 2000 [9,10]. Since the risk of polio importation and re-emergence persists, it is important to maintain a high population level of immunity and surveillance quality to mitigate the risks of polio outbreaks. Since the vaccination coverage for polio in Korea has been maintained above 95% [11], this study focused on understanding the status of AFP surveillance and evaluating the quality of surveillance to prevent the re-emergence of polio in Korea.

During 2002–2011 in Korea, the non-polio AFP rate was only average at 0.33 (range 0.10–0.89) per 100,000 population among people aged under 15 years [5]. However, the annualized non-polio AFP rate had been maintained at ≥1.0 since 2012, and adequate stool specimen collection was continually achieved at ≥80% of AFP cases since 2002. Although the quality of the AFP surveillance system at the national level was increased considerably during the study period compared to before 2012, the non-polio AFP rate was below the standard 1.0 in some provinces in Korea. Therefore, it is necessary to further cooperate with the regional participating hospitals to improve the sensitivity of AFP surveillance.

NPEVs are known to be related to AFP and may become more relevant as major causative agents of AFP at the polio eradication stage [12,13,14,15,16]. In this study, EV-A71 was the most frequently detected NPEV type and had a significant impact on the overall detection rate of NPEV in the AFP surveillance in Korea. In other countries, especially in the Asia region, the most detected NPEV in the AFP surveillance is also EV-A71 [16]. According to a previous study, EV-A71 was consistently reported as a major circulating NPEV type during 1999–2019 in Korea, and it is associated with both mild syndromes (herpangina and HFMD) and serious neurological diseases [8]. Although the pathogenic mechanism of EV-A71 is still not fully understood, several amino acid positions have been identified as virulent determinants of EV-A71 [17,18,19,20,21].

Acute flaccid myelitis (AFM) is a serious neurologic disease that causes muscle weakness and paralysis similar to poliomyelitis. In recent years, EV-D68-associated AFM outbreaks have been reported in several countries [22,23,24]. EV-D68 was first identified in 1962 and is involved in causing mild respiratory diseases [22,23]. A previous study reported that EV-D68 isolates from the AFM outbreak in 2014 acquired unique substitutions related to the potential neuropathology and evolved into a distinct phylogenetic lineage [25]. Reports on the identification of NPEV from neurological cases have raised concerns that spontaneous mutations and recombination in NPEV may lead to an outbreak of severe diseases with neurological symptoms. During the study period, EV-D68 was not identified during AFP surveillance in Korea due to the limitation related to the stool specimen collection for AFP surveillance. Further analysis of other specimens such as the cerebrospinal fluid or respiratory specimen will be required for the investigation of NPEV-related neurological diseases.

Virus isolation in cell culture assay has been the gold standard for the detection of enteroviruses as well as poliovirus [7]. However, EV-A is known to be difficult to isolate in cell culture because certain EVs, especially group A coxsackievirus, adapt poorly to the cells [26]. Our study indicated that the real-time RT-PCR and conventional RT-PCR method might be more useful in AFP surveillance as it is more sensitive for detecting viruses compared to virus isolation assay in cell culture.

AFP surveillance is one of the important projects for global polio eradication, but more intensive monitoring and genetic variation analysis for NPEV may be helpful in preparing for the outbreak of infectious diseases, including those with high disease burdens such as polio.

## Figures and Tables

**Table 1:** Classification of AFP patient surveillance indicators in Korea, 2012–2019.

	2012	2013	2014	2015	2016	2017	2018	2019
No. of annual expected cases in children <15 years of age	76	76	72	70	69	68	67	66
Reported number of cases	94	83	88	83	69	68	70	82
Non-polio AFP rate	1.24	1.11	1.22	1.19	1.00	1.00	1.04	1.21
Adequate specimen (%)	89	94	86	86	94	94	90	85
60-day follow-up (%)	100	100	100	100	100	100	100	100

AFP, acute flaccid paralysis.

**Table 2:** Sex, age, fever at the onset, and IPV distribution among AFP patients in Korea surveillance, 2012–2019.

	2012 *n* = 94	2013 *n* = 83	2014 *n* = 88	2015 *n* = 83	2016 *n* = 69	2017 *n* = 68	2018 *n* = 70	2019 *n* = 82	Total *n* = 637	*p*-Value
Sex										0.0009
Male	54 (57.4)	48 (57.8)	42 (47.7)	49 (59.0)	46 (66.7)	41 (60.3)	40 (57.1)	51 (62.2)	371 (58.2)	
Female	40 (42.6)	35 (42.2)	46 (52.3)	34 (41.0)	23 (33.3)	27 (39.7)	30 (42.9)	31 (37.8)	266 (41.8)	
Age, years										<0.0001
<1	6 (6.4)	7 (8.4)	3 (3.4)	6 (7.2)	2 (2.9)	3 (4.4)	1 (1.4)	3 (3.7)	31 (4.9)	
1–5	65 (69.1)	44 (53.0)	46 (52.3)	50 (60.2)	41 (59.4)	24 (35.3)	25 (35.7)	38 (46.3)	333 (52.3)	
6–10	10 (10.6)	17 (20.5)	23 (26.1)	14 (16.9)	15 (21.7)	26 (38.2)	23 (32.9)	24 (29.3)	152 (23.9)	
11–15	13 (13.8)	13 (15.7)	14 (15.9)	13 (15.7)	9 (13.0)	15 (22.1)	18 (25.7)	17 (20.7)	112 (17.6)	
>15	0 (0.0)	3 (3.6)	2 (2.3)	0 (0.0)	2 (2.9)	0 (0.0)	3 (4.3)	0 (0.0)	10 (1.6)	
Fever at onset										0.0001
Yes	65 (69.1)	57 (68.7)	58 (65.9)	62 (74.7)	42 (60.9)	35 (51.5)	37 (52.9)	53 (64.6)	409 (64.2)	
No	29 (30.9)	26 (31.1)	30 (34.1)	21 (25.3)	27 (39.1)	33 (48.5)	33 (47.1)	29 (35.4)	228 (35.8)	
IPV dose										<0.0001
0	2 (2.1)	3 (3.6)	5 (5.7)	3 (3.6)	0 (0.0)	0 (0.0)	1 (1.4)	1 (1.2)	15 (2.4)	
1	1 (1.1)	2 (2.4)	3 (3.4)	1 (1.2)	2 (2.9)	0 (0.0)	0 (0.0)	2 (2.4)	11 (1.7)	
2	3 (3.2)	2 (2.4)	2 (2.3)	3 (3.6)	0 (0.0)	1 (1.5)	1 (1.4)	0 (0.0)	12 (1.9)	
3	25 (26.6)	37 (44.6)	30 (34.1)	34 (41.0)	20 (29.0)	18 (26.5)	16 (22.9)	33 (40.2)	213 (33.4)	
4	29 (30.9)	19 (22.9)	32 (36.4)	28 (33.7)	33 (47.8)	33 (48.5)	26 (37.1)	27 (32.9)	227 (35.6)	
5	1 (1.1)	0 (0.0)	0 (0.0)	0 (0.0)	0 (0.0)	0 (0.0)	0 (0.0)	0 (0.0)	1 (0.2)	
Unknown	33 (35.1)	20 (24.1)	16 (18.2)	14 (16.9)	14 (20.3)	16 (23.5)	26 (37.1)	19 (23.2)	158 (24.8)	

IPV, inactivated polio vaccine; AFP, acute flaccid paralysis.

**Table 3:** Virus detection during AFP surveillance in Korea, 2012–2019.

Year	Total no. of AFP Patientswith Specimens	Virus Isolation Results			Real-Time RT-PCR Positive	Negative
		L20B Positive	L20B Positive + NPEV	RD-A Positive		
2012	94	0	0	8	34	60
2013	83	0	0	2	47	36
2014	88	0	1	8	41	47
2015	83	0	0	8	30	53
2016	69	0	0	7	33	36
2017	68	0	0	4	6	62
2018	70	0	0	5	6	64
2019	82	0	1	7	16	66
Total	637	0	2	49	213	424

AFP, acute flaccid paralysis; L20B, murine cells; RD-A, rhabdomyosarcoma; RT-PCR, reverse transcription polymerase chain reaction.

**Table 4:** Distribution of NPEV genotypes of AFP surveillance in Korea, 2012–2019.

NPEV-Types		No. of Viral RNA Detected										No. of Virus Isolation
		2012 (*n* = 94)	2013 (*n* = 83)	2014 (*n* = 88)	2015 (*n* = 83)	2016 (*n* = 69)	2017 (*n* = 68)	2018 (*n* = 70)	2019 (*n* = 82)	Total (*n* = 637)	Rate (%)	
**Enterovirus A**		28	35	30	16	21	2	1	13	146	68.5	26
	CA2	0	0	3	0	1	0	0	0	4	1.9	1
	CA4	1	0	2	0	1	0	0	0	4	1.9	0
	CA5	0	1	0	0	0	1	0	3	5	2.3	2
	CA6	4	0	0	0	0	0	0	0	4	1.9	0
	CA10	0	2	0	1	0	0	0	1	4	1.9	2
	CA16	0	0	4	3	0	0	0	1	8	3.8	4
	EV-A71	23	32	21	12	19	1	1	8	117	54.9	17
**Enterovirus B**		6	5	6	2	6	3	5	2	35	16.4	18
	CA9	0	0	0	0	0	0	0	1	1	0.5	0
	CB1	0	0	0	1	0	0	0	0	1	0.5	0
	CB2	0	0	0	0	0	1	0	0	1	0.5	1
	CB3	2	0	0	0	0	0	0	1	3	1.4	2
	CB5	0	1	6	0	0	0	0	1	8	3.8	6
	E3	0	0	0	0	0	1	1	0	2	0.9	3
	E6	1	0	0	1	0	0	0	0	2	0.9	0
	E7	2	0	0	0	0	0	0	0	2	0.9	2
	E11	0	0	0	0	1	0	0	0	1	0.5	0
	E12	0	0	0	0	2	1	0	0	3	1.4	0
	E13	0	0	0	0	0	0	1	0	1	0.5	1
	E18	0	0	0	0	3	0	0	0	3	1.4	0
	E30	1	4	0	0	0	0	1	0	6	2.8	3
**Enterovirus C**		0	0	0	0	1	1	0	0	2	0.9	0
	CA19	0	0	0	0	1	0	0	0	1	0.5	0
	CA24	0	0	0	0	0	1	0	0	1	0.5	0
**UT**		0	7	5	12	5	0	0	1	30	14.1	5
**Total**		34	47	41	30	33	6	6	16	213	100	49
**EV detection rate (%)**		36.2	56.6	46.6	36.1	47.8	8.8	8.6	19.5	33.4	-	7.7

Abbreviation: UT, untypable; AFP, acute flaccid paralysis; NPEV, non-polio enterovirus.

## Data Availability

The data presented in this study are available in this article.
